# Detailed kinetics and regulation of mammalian 2-oxoglutarate dehydrogenase

**DOI:** 10.1186/1471-2091-12-53

**Published:** 2011-09-26

**Authors:** Feng Qi, Ranjan K Pradhan, Ranjan K Dash, Daniel A Beard

**Affiliations:** 1 Biotechnology and Bioengineering Center and Department of Physiology, Medical College of Wisconsin, Milwaukee, WI-53226, USA; 2 Sanford-Burnham Medical Research Institute at Lake Nona, 6400 Sanger Road, Orlando, FL, 32827, USA

## Abstract

**Background:**

Mitochondrial 2-oxoglutarate (α-ketoglutarate) dehydrogenase complex (OGDHC), a key regulatory point of tricarboxylic acid (TCA) cycle, plays vital roles in multiple pathways of energy metabolism and biosynthesis. The catalytic mechanism and allosteric regulation of this large enzyme complex are not fully understood. Here computer simulation is used to test possible catalytic mechanisms and mechanisms of allosteric regulation of the enzyme by nucleotides (ATP, ADP), pH, and metal ion cofactors (Ca^2+ ^and Mg^2+^).

**Results:**

A model was developed based on an ordered ter-ter enzyme kinetic mechanism combined with con-formational changes that involve rotation of one lipoic acid between three catalytic sites inside the enzyme complex. The model was parameterized using a large number of kinetic data sets on the activity of OGDHC, and validated by comparison of model predictions to independent data.

**Conclusions:**

The developed model suggests a hybrid rapid-equilibrium ping-pong random mechanism for the kinetics of OGDHC, consistent with previously reported mechanisms, and accurately describes the experimentally observed regulatory effects of cofactors on the OGDHC activity. This analysis provides a single consistent theoretical explanation for a number of apparently contradictory results on the roles of phosphorylation potential, NAD (H) oxidation-reduction state ratio, as well as the regulatory effects of metal ions on ODGHC function.

## Background

The 2-oxoglutarate (α-ketoglutarate; αKG) dehydrogenase complex (OGDHC, EC 1.2.4.2, EC 2.3.1.61, and EC 1.6.4.3) is a multi-enzyme complex which catalyzes the chemical reaction:

(1) $$\begin{array}{c} \alpha \text{K}{\text{G}}^{\text{2 - }}\text{ + CoAS}{\text{H}}^{4-}\text{ + NA}{\text{D}}^{-}+{\text{H}}_{\text{2}}\text{O}⇌\\ \text{Succinyl - Co}{\text{A}}^{4-}{\text{ + CO}}_{\text{3}}^{\text{2}-}\text{ + NAD}{\text{H}}^{\text{2}-}\text{ + }{\text{H}}^{\text{ + }} \end{array}$$

**Figure 1:**
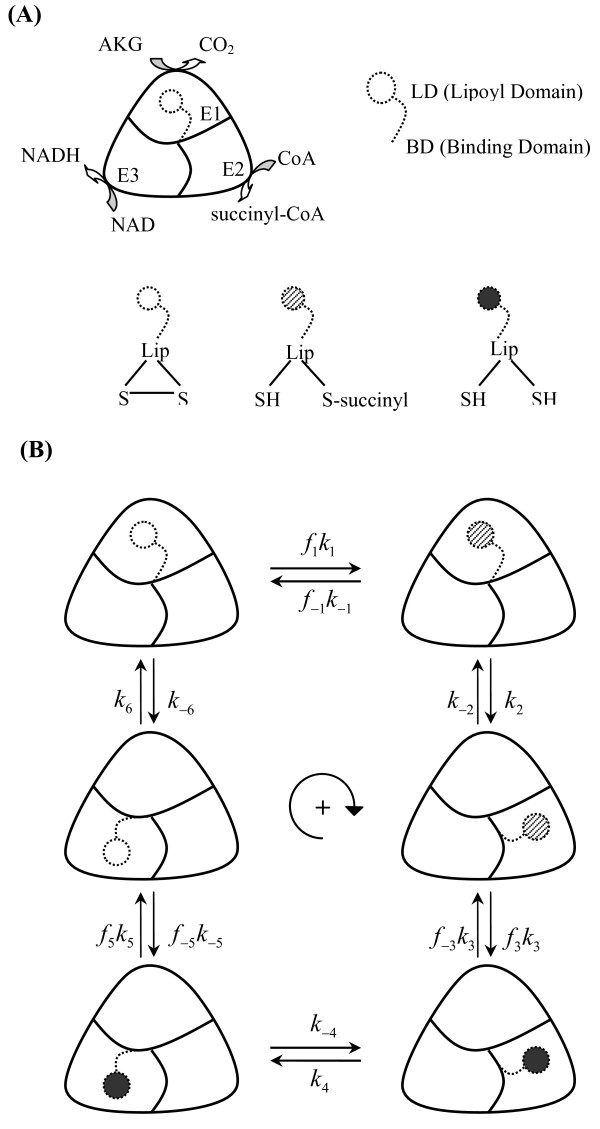
**Schematic representation of the proposed mechanism of 2-oxoglutarate dehydrogenase complex (OGDHC)**. (A) It consists of three component enzymes: oxoglutarate dehydrogenase (E1), dihydro-lipoamide succinyltransferase (E2), and dihydro-lipoamide dehydrogenase (E3). The schematic representation here does not describe true stoichiometry of the multiple copies of three enzymes in the complex. The binding domain and lipoyl domain of E2 polypeptide are connected to the complex core with flexible links (dotted lines), are used here to describe the mechanism in which a single lipoic acid rotates between the three catalytic sites. In the catalytic cycle, the disulfide at the tip of the lipoyl can be in oxidized, reduced or semi-reduced lipoate forms, the later one is connected with succinyl residue transferred from oxoglutarate. (B) This schematic illustrates the proposed kinetic schemes along with the mechanism of conformational changes. The forward reaction is read in the clockwise direction. The complex has three binding sites: one for each composite enzyme. E1 binds to 2-oxoglutarate or corresponding product CO_2 _(top), E2 binds to COA or corresponding product Succinyl-CoA (bottom-right), E3 binds to NAD or corresponding product NADH (bottom-left). It is assumed that, in the process of conformational changes, the rotation of one lipoic acid between three catalytic sites leads to transfer succinyl from E1 to E2 and proton from E2 to E3.

OGDHC is primarily located within the mitochondrial matrix and is a key regulatory enzyme complex in the TCA cycle, responsible for oxidative decarboxylation of 2-oxoglutarate, transferring a succinyl group to coenzyme A (CoASH^4-^) and producing reducing equivalents (NADH^2-^) for the electron transport system. Regulation of OGDHC not only affects the distribution of 2-oxoglutarate between the TCA cycle and malate-aspartate shuttle system, but also has effects on the oxidative deamination of glutamate. OGDHC is a crucial target of reactive oxygen species (ROS) and also able to generate ROS, which make it distinctly important for bioenergetics [1]. The molecular organization of OGDHC is similar to that of the pyruvate dehydrogenase complex (PDHC) as it belongs to the same heterogeneous family of 2-oxo acid dehydrogenase multi-enzyme complexes [2]. It consists of multiple copies of three enzyme components: oxoglutarate dehydrogenase (E1), dihydro-lipoamide succinyltransferase (E2), and dihydro-lipoamide dehydrogenase (E3). Consecutive actions of these enzymes catalyze the oxidation of 2-oxoglutarate and reduction ofNAD^-^, which results in the production of NADH^2- ^and Succinyl-CoA^4- ^(Figure 1A). Allosteric interactions associated with the E1 component are known to be the predominant target for controlling of OG-DHC activity [3].

OGDHC was first purified from the pig heart mitochondria by Sanadi *et al*. [4] and subsequently studied by many researchers to examine its catalytic and regulatory properties within permeabilized, un-coupled, and coupled mitochondria from a variety of mammalian tissues [5-11]. A catalytic mechanism for the overall reaction of the enzyme complex was also first proposed by Sanadi *et al*. [4] which suggested that the coenzyme, NAD^-^, and 2-oxoglutaric acid participate in the reaction with the help of the cofactors thiamine pyrophosphate (TPP), lipoic acid, and FAD^2- ^[12,13]. Their proposed mechanism is a Hexa-Uni-Ping-Pong mechanism in Cleland's terminology [14] where it is assumed that the first product (CO_2_) is released before the second substrate (CoASH^4-^) binds, and the second product (Succinyl-CoA^4-^) is released before the third substrate (NAD^-^) binds to the enzyme. Subsequently, Koike *et al*. [15] postulated another mechanism in which, the lipoic acids transfer intermediates by rotating between the three catalytic sites. Furthermore, experimental results of fluorescence resonance energy transfer and dynamic anisotropy showed that the lipoic acids in the E2 component undergo motion where they rotate between different catalytic sites [16-18]. The results of steady-state kinetic studies done by Hamada *et al*. [19] and Smith *et al*. [10] contradict each other, and not all results are compatible with the Sanadi mechanism [4]. This issue was addressed by Mcminn and Ottaway [20] with kinetic studies based on the Fromm method [21]. Mcminn and Ottaway [20] explained the observed nonlinearity in the reciprocal plots of the results and proposed a phenomenological mechanism with semi-random characteristic. A recent study by Aevarsson *et al*. [22] on the crystal structure and architecture of 2-oxo acid dehydrogenase multi-enzyme complexes, provides interesting insights into the plausible kinetic mechanism of 2-oxo acid dehydrogenase family which includes OGDHC.

It has been consistently shown that the activity of OGDHC is controlled by various factors, including the variations of the NAD oxidation-reduction state, the state of phosphorylation of the nucleotide systems, and the ratio of succinyl-CoA to CoA-SH. Regulation by reversible phosphorylation has not been demonstrated. Experiments in isolated mitochondria of heart, liver, and kidney have shown that the OGDHC is regulated by Ca^2+ ^ions with a marked decrease in the apparent *K_m _*for 2-oxoglutarate in the presence of adenine nucleotides and minimal effect of Ca^2+ ^at saturating concentration of 2-oxoglutarate [9,23-25]. Moreover, the apparent *K_m _*for 2-oxoglutarate is lowered by a decrease in the ATP/ADP ratio, which can significantly increase the sensitivity of the enzyme to Ca^2+^ions [23,25]. It has been reported that the maximum activity of OGDHC is unaffected by changes in pH, while the apparent *K_m _*of the enzyme for 2-oxoglutarate is greatly altered by changes in pH over the range of 6.5 - 7.5 [23]. A number of studies have also demonstrated the possible role of Mg^2+ ^ions in the regulation of OGDHC either by directly affecting the activity of the enzyme or by modulating the Ca^2+ ^effect on the enzyme. Mg^2+ ^ion has been shown to increase [26-28] or to have no effect on the activity of OGDHC [29,30]. McCormack and Denton [23] studied isolated OGDHC from pig heart mitochondria and found that there is no effect of EDTA and 1 mM Mg^2+ ^on the activity of OGDHC when Ca^2+ ^concentration was effectively less that 1 μM. Panov and Scarpa [9] concluded that the effects of Mg^2+ ^and Ca^2+ ^ions on the OGDHC activity are additive only at relative low concentration of free cations which suggested that at high concentrations, each ion may compete each other for binding sites. It is also evident that, in the presence of low Ca^2+ ^concentration, Mg^2+ ^ion can strongly modify the enzyme's affinities for 2-oxoglutarate and NAD^-^[9]. However, the kinetic mechanisms by which these divalent metal ions regulate the properties of mitochon-drial OGDHC are not understood.

Although a number of attempts have been made to understand the catalytic mechanisms of OGDHC, both experimentally and theoretically, there is no mechanistic model that consistently explains the available experimental data on the kinetics of this enzyme complex and adequately describes the regulatory roles of nucleotides and other metal ion cofactors (Ca^2+^, Mg^2+^, etc.). Therefore a mechanistic model of OGDHC is needed to understand the orchestrated controlling of OGDHC by cofactors inside mitochondria under different physiological conditions. In the present work, a kinetic model of OGDHC is introduced to quantitatively understand the catalytic properties and regulation of OGDHC, based on the observations from a large number of independent experimental studies in mammalian tissues. The model accurately describes the catalytic properties of this enzyme complex observed experimentally, and clarifies many contradictory results reported in earlier studies.

## Methods

In this section, we first present a general kinetic model for conformational changes in OGDHC, based on a presumed ter-ter enzyme mechanism via substrate channeling. The model is then used to characterize the kinetics of the 2-oxoglutarate dehydrogenase reaction (Equation 1) and further extended to describe the regulatory roles of cofactors, i.e., nucleotides and various metal ions. The kinetic parameters of the model are estimated using a wide variety of experimental data, available in the literature.

### Kinetic scheme for conformational changes in a ter-ter enzyme mechanism

The kinetic equation of the proposed model for OGDHC reaction is derived from a ter-ter enzyme me-chanism combined with a model of conformational changes that represent the rotation of the single lipoic acid between different catalytic sites [15,18]. The derivation is inspired by a previously developed model for trans-carboxylase [31]. The assumption of the model is that the enzyme complex is composed of three sub-enzyme (E1, E2, and E3), each with one binding site: site 1 binds to 2-oxoglutarate (*α*KG^2-^) or corresponding product CO_2_, site 2 binds to CoASH^4- ^or corresponding product Succinyl-CoA^4-^, site 3 binds toNAD^- ^or corresponding product NADH^2- ^(Figure 1A). Furthermore, the basic mechanism involves conformational changes, where the rotation of one lipoic acid between three catalytic sites leads to transfer of succinyl from E1 to E2 and proton from E2 to E3. In the catalytic cycle, the disulfide at the tip of the lipoyl can be in oxidized, reduced or semi-reduced li-poate forms, the semi-reduced form is bound with succinyl residue transferred from 2-oxoglutarate (Figure 1B).

Each of the six conformational states shown in Figure 1B can involve any possible binding states associated with the enzyme. For example, the first site is either empty or bound to 2-oxoglutarate or CO_2_; the second site is either empty or bound to CoASH^4- ^or Succinyl-CoA^4-^; and the third site is either empty or bound to NAD^- ^or NADH^2-^. Therefore, there are a total of 27 binding states for each one of the six conformational states, which gives rise to 27 × 6 = 162 distinct states in the model. Here we denote these 162 states as $E_{xyz}^{i}$, where *i *∈ {1, 2, 3, 4, 5, 6} represents the index for conformational states, and *x *∈ {Ø, A, P}, *y *∈ {Ø, B, Q} and *z *∈ {Ø, C, R} represent the binding states of site 1, site 2, and site 3. The lower-case $e_{xyz}^{i}$ is used to represent the fraction of each state. Therefore, the total fractional states can be expressed as

(2) $$e_{total}^{i}=\underset{x\in \left\{\emptyset ,\text{A,P}\right\},y\in \left\{\emptyset ,\text{B,Q}\right\},z\in \left\{\emptyset ,\text{C,R}\right\}}{\sum }e_{xyz}^{i}.$$

We assume rapid equilibrium binding for all 27 binding states, implying that the binding processes are much faster than the conformational change processes. With this assumption, Equation (2) can be written as:

(3) $$\begin{array}{ccc} e_{total}^{i} & =e_{free}^{i}\times \left(1+\left[\text{A}\right]∕K_{A}+\left[\text{P}\right]∕K_{P}\right) & \\ & \times \left(1+\left[\text{B}\right]∕K_{B}+\left[\text{Q}\right]∕K_{Q}\right)\\ & \times \left(1+\left[\text{C}\right]∕K_{C}+\left[\text{R}\right]∕K_{R}\right), \end{array}$$

where $e_{free}^{i}\equiv e_{\emptyset \emptyset \emptyset }^{i}$ denotes the fraction of free enzyme complex that binds to the reactants; *K_A_, K_B_, K_C_, K_P_, K_Q _*and *K_R _*are the dissociation constants associated with the binding of reactants (A: *α*KG^2-^, B: CoASH^4- ^and C: NAD^-^) and products (P: CO_2_, Q: Succinyl-CoA^4- ^and R: NADH^2-^) to the enzyme complex. Here, we assumed that these constants do not depend on conformational states of the enzyme complex and the bound reactant at one site does not influence the binding reaction at another site: all binding interactions are independent of one other. These assumptions are necessary to make the model tractable, and are validated by comparing the model predictions to the available experimental data.

We define *f_i _*as the fractions in conformation state *i *(*i *∈ {1, 3, 5}) which can undergo forward conformational transformation to state *i*+1 (lipoate changes in three different redox forms). Similarly, *f*_-*i *_is the fraction in conformation state *i*+1 which can undergo conformational transformation in the reverse direction (Figure 1B). Specifically, the binding of 2-oxoglutarate at site 1 is necessary for transition from conformation state 1 to 2. Therefore, we can write

(4) $$\begin{array}{c} f_{1}=\left(\frac{\left[\text{A}\right]∕K_{A}}{1+\left[\text{A}\right]∕K_{A}+\left[\text{P}\right]∕K_{P}}\right),\\ f_{\text{ - 1}}=\left(\frac{\left[\text{P}\right]∕K_{P}}{1+\left[\text{A}\right]∕K_{A}+\left[\text{P}\right]∕K_{P}}\right),\\ f_{3}=\left(\frac{\left[\text{B}\right]∕K_{B}}{1+\left[\text{B}\right]∕K_{B}+\left[\text{Q}\right]∕K_{Q}}\right),\\ f_{\text{ - 3}}=\left(\frac{\left[\text{Q}\right]∕K_{Q}}{1+\left[\text{B}\right]∕K_{B}+\left[\text{Q}\right]∕K_{Q}}\right),\\ f_{5}=\left(\frac{\left[\text{C}\right]∕K_{C}}{1+\left[\text{C}\right]∕K_{C}+\left[\text{R}\right]∕K_{R}}\right),\\ f_{\text{ - 5}}=\left(\frac{\left[\text{R}\right]∕K_{R}}{1+\left[\text{C}\right]∕K_{C}+\left[\text{R}\right]∕K_{R}}\right). \end{array}$$

The net turn-over (reaction velocity) for this mechanism can be expressed

(5) $$v=\frac{V}{\left[E_{total}\right]}=f_{5}k_{5}\left[e_{total}^{5}\right]-f_{-5}k_{-5}\left[e_{total}^{6}\right].$$

Applying the King and Altman method to the scheme shown in Figure 1B gives the following expression for the net reaction velocity:

(6) $$v=\frac{f_{1}f_{3}f_{5}k_{1}k_{2}k_{3}k_{4}k_{5}k_{6}-f_{-1}f_{-3}f_{-5}k_{-1}k_{-2}k_{-3}k_{-4}k_{-5}k_{-6}}{\left(\begin{array}{c} f_{-1}f_{-3}k_{-1}k_{-2}k_{-3}k_{-4}\left(k_{6}+k_{-6}\right)+f_{-1}f_{5}k_{-1}k_{-2}k_{4}k_{5}\left(k_{6}+k_{-6}\right)\\ +f_{3}f_{5}k_{2}k_{3}k_{4}k_{5}\left(k_{6}+k_{-6}\right)+f_{-3}f_{-5}\left(k_{2}+k_{-2}\right)k_{-3}k_{-4}k_{-5}k_{-6}\\ +f_{-3}f_{1}\left(k_{2}+k_{-2}\right)k_{-3}k_{-4}k_{1}k_{6}+f_{1}f_{5}\left(k_{2}+k_{-2}\right)k_{1}k_{4}k_{5}k_{6}\\ +f_{1}f_{3}\left(k_{4}+k_{-4}\right)k_{1}k_{2}k_{3}k_{6}+f_{-1}f_{-5}\left(k_{4}+k_{-4}\right)k_{-5}k_{-6}k_{-1}k_{-2}\\ +f_{3}f_{-5}\left(k_{4}+k_{-4}\right)k_{-5}k_{-6}k_{2}k_{3}+f_{-1}f_{-3}f_{5}k_{-1}k_{-3}k_{5}k_{-2}\left(k_{6}+k_{-6}\right)\\ +f_{-1}f_{3}f_{5}k_{-1}k_{3}k_{5}k_{4}\left(k_{6}+k_{-6}\right)+f_{-3}f_{-5}f_{1}k_{-3}k_{-5}k_{1}k_{-4}\left(k_{2}+k_{-2}\right)\\ +f_{-3}f_{1}f_{5}k_{-3}k_{1}k_{5}k_{6}\left(k_{2}+k_{-2}\right)+f_{-1}f_{-5}f_{3}k_{-1}k_{-5}k_{3}k_{-6}\left(k_{-4}+k_{4}\right)\\ +f_{-5}f_{1}f_{3}k_{-5}k_{1}k_{3}k_{2}\left(k_{4}+k_{-4}\right)+f_{1}f_{3}f_{5}k_{1}k_{3}k_{5}\left(k_{4}k_{2}+k_{6}k_{4}+k_{2}k_{6}\right)\\ +f_{-1}f_{-3}f_{-5}k_{-1}k_{-3}k_{-5}\left(k_{-2}k_{-4}+k_{-4}k_{-6}+k_{-6}k_{-2}\right) \end{array}\right)}.$$

This complex expression can also be obtained using our KAPattern package [32], available freely for the derivation of enzyme rate equations. Substituting the fractional occupancy distributions as defined in Equation (4), we obtain an expression for the reaction velocity in terms of the individual rate constants and dissociation constants. The kinetic constants can be also expressed in terms of various rate constants. Using the Haldane relationship, the velocity equation can be written as:

(7) $$v=\frac{V_{f}V_{r}\left([\text{A][B][C}]-[\text{P][Q][R}]/K_{eq}\right)}{\left(\begin{array}{l} V_{r}[\text{A][B][C}]+K_{mC}V_{r}[\text{A][B][R}]/K_{ir}\\ +K_{mC}V_{r}[\text{A][B]+}K_{mB}V_{r}[\text{A][C][Q}]/K_{iq}\\ +K_{mB}V_{r}[\text{A][C}]+K_{mP}V_{f}[\text{A][Q][R}]/K_{ia}/K_{eq}\\ +K_{ic}K_{mB}V_{r}[\text{A][Q]/}K_{iq}\text{+}K_{mA}V_{r}[\text{B][C][P}]/K_{ip}\\ +K_{mA}V_{r}\text{[B][C}]+K_{ia}K_{mC}V_{r}[\text{B][P][R}]/K_{ip}/K_{ir}\\ +K_{ia}K_{mC}V_{r}\text{[B][R}]/K_{ir}+K_{ib}K_{mA}V_{r}[\text{C][P][Q}]/K_{iq}/K_{ip}\\ +K_{ib}K_{mA}V_{r}[\text{C][P]/}K_{ip}\text{+}V_{f}\text{[P][Q][R}]/K_{eq}\\ +K_{mR}V_{f}[\text{P][Q]/}K_{eq}\text{+}K_{mQ}V_{f}[\text{P][R}]/K_{eq}+K_{mP}V_{f}\text{[Q][R}]/K_{eq} \end{array}\right)},$$

where the kinetic constants are defined as:

$$\begin{array}{c} V_{f}=num_{1}∕Coef_{ABC},\;V_{r}=num_{2}∕Coef_{PQR},\;K_{mA}=Coef_{BC}∕Coef_{ABC},\;K_{mB}=Coef_{AC}∕Coef_{ABC},\\ K_{mC}=Coef_{AB}∕Coef_{ABC},\;K_{mP}=Coef_{QR}∕Coef_{PQR},\;K_{mQ}=Coef_{PR}∕Coef_{PQR},\;K_{mR}=Coef_{PQ}∕Coef_{PQR},\\ K_{ip}=Coef_{BR}∕Coef_{BPR}=Coef_{BC}∕Coef_{BCP},\;K_{iq}=Coef_{CP}∕Coef_{CPQ}=Coef_{AC}∕Coef_{ACQ},\\ K_{ir}=Coef_{AQ}∕Coef_{AQR}=Coef_{AB}∕Coef_{ABR},\;K_{ia}=Coef_{QR}∕Coef_{AQR}=Coef_{BR}∕Coef_{ABR},\\ K_{ib}=Coef_{PR}∕Coef_{BPR}=Coef_{CP}∕Coef_{BCP},\;K_{ic}=Coef_{PQ}∕Coef_{CPQ}=Coef_{AQ}∕Coef_{ACQ}\;\text{and}\;\\ K_{eq}=num_{1}∕num_{2}. \end{array}$$

Here we used the shorthand notation similar to that of Segel [33] where *num *= *k*_1 _*k*_2 _*k*_3 _*k*_4 _*k*_5 _*k_6_K_P_K_Q_K_R_, num *= *k*_-1 _*k*_-2 _*k*_-3 _*k*_-4 _*k*_-5 _*k*_-6_*K_A_K_B_K_C_*, and *Coef_AB _= K_C_K_P_K_Q_K_R _*(*k*_1 _*k*_2 _*k*_3 _*k*_6 _(*k*_4 _+ *k*_-4_)), and so on. *V_f _*and *V_r _*have units of mass per unit time per mass of protein. Other kinetic parameters associated with the binding of reactants and products have the units of concentration (mass per unit volume).

In the ter-ter biochemical reaction, the fourteen unknown kinetic parameters in Equation (7) are related to the equilibrium constant *K_eq _*(known) via the following equilibrium relationship:

(8) $$\begin{array}{ccc} K_{eq} & ={\left(\frac{\text{[P][Q][R]}}{\text{[A][B][C]}}\right)}_{\text{eq}}=\frac{num_{1}}{num_{2}} & \\ & =\frac{K_{ip}K_{iq}K_{ir}}{K_{ia}K_{ib}K_{ic}}=\frac{V_{f}K_{mP}K_{iq}K_{ir}}{V_{r}K_{ia}K_{mB}K_{ic}}\\ & =\frac{V_{f}K_{ip}K_{mQ}K_{ir}}{V_{r}K_{ia}K_{ib}K_{mC}}=\frac{V_{f}K_{ip}K_{iq}K_{mR}}{V_{r}K_{mA}K_{ib}K_{ic}}, \end{array}$$

reducing the total number of independent unknown kinetic parameter to thirteen.

### Kinetic model of OGDHC using a ter-ter enzyme mechanism

We apply the above general form of the ter-ter enzyme mechanism for the analysis of available expe-rimental data on the kinetic of OGDHC to estimate the unknown kinetic parameters and to elucidate whether the proposed mechanism is able to explain the available kinetic data.

In the TCA cycle, OGDHC is primarily involved in the fifth step for oxidation of acetyl-CoA. The reference chemical reaction is given by Equation (1). The corresponding biochemical reaction is given by

(9) $$\begin{array}{c} \alpha \text{KG + CoASH }\\ \text{ + NAD}⇌\text{succinyl}-\;{\text{CoA + CO}}_{\text{2,tot}}^{}\text{ + NADH}\text{.} \end{array}$$

Here biochemical reactants, such as *α*KG, correspond to ensemble chemical species, such as *α*KG^2-^, H *α*KG^-^, etc. The chemical reaction in Equation (1) is unambiguously balanced in terms of mass and charge, whereas this biochemical reaction is not. In this reaction, the reactant CO_2, tot _represents the sum of aqueous carbon dioxide and bicarbonate species (${\text{CO}}_{3}^{\text{2}-}$, ${\text{HCO}}_{\text{3}}^{-}$ and H_2_CO_3_).

The equilibrium constant for the reference reaction can be written as:

(10) $$\begin{array}{ccc} K_{eq,ogdhc}^{0} & ={\left(\frac{\text{[Succinyl - Co}{\text{A}}^{4-}{\text{][CO}}_{\text{3}}^{\text{2}-}\text{][NAD}{\text{H}}^{\text{2}-}\text{][}{\text{H}}^{\text{ + }}\text{]}}{\text{[}\alpha \text{K}{\text{G}}^{\text{2 - }}\text{][CoAS}{\text{H}}^{4-}\text{][NA}{\text{D}}^{-}\text{]}}\right)}_{eq} & \\ & =\exp\left(-\frac{\Delta_{r}G_{ogdhc}^{0}}{RT}\right), \end{array}$$

where $\Delta_{r}G_{ogdhc}^{0}$ is the standard Gibbs free energy of the reference reaction which is computed using the basic thermodynamic data (298.15 K, I = 0.15 M) listed in Li *et al*. [34].

For the development of the kinetic model of OGDHC, we assume that the ter-ter enzyme mechanism proposed in the previous section along with the conformational changes (Figure 1B) can explain the observed kinetics of OGDHC. Because the kinetic data we used here to estimate the unknown kinetic parameters were all from the initial velocity studies in which only the products NADH^2- ^and Succinyl-CoA^- ^were present in the reaction mediums, the forward flux of OGDHC can be reduced from Equation (7) as:

(11) $$J_{ogdhc}^{+}=\frac{V_{f}[\text{A][B][C}]}{\left(\begin{array}{l} {}[\text{A][B][C}]+K_{mC}[\text{A][B][R}]/K_{ir}\\ +K_{mC}[\text{A][B]+}K_{mB}[\text{A][C][Q}]/K_{iq}\\ +K_{mB}[\text{A][C}]+K_{ic}K_{mB}[\text{A][Q]/}K_{iq}\\ +K_{mB}K_{ic}[\text{A][Q][R}]/K_{iq}/K_{ir}\\ +K_{mA}\text{[B][C}]+K_{ia}K_{mC}\text{[B][R}]/K_{ir}\\ +K_{mB}K_{ic}K_{ia}\text{[Q][R}]/K_{iq}/K_{ir} \end{array}\right)},$$

where [A], [B], [C], [R] and [Q] represent the concentrations of *α*KG^2-^, CoASH^4-^, NAD^-^, NADH^2-^, and Succinyl-CoA^4-^, respectively. This kinetic expression for OGDHC reaction contains 8 unknown kinetic parameters. Thus, this expression was used first to estimate the 8 unknown kinetic parameters. Using the relationship $J_{ogdhc}^{+}∕J_{ogdhc}^{-}=\exp\left(\Delta G∕RT\right)$ to determine the reverse flux [35], we obtain the full flux expression for OGDHC as:

(12) $$\begin{array}{ccc} J_{ogdhc}^{} & =J_{ogdhc}^{+}-J_{ogdhc}^{-} & \\ & =J_{ogdhc}^{+}\left(1-\frac{1}{K_{eq,ogdhc}^{0}}\frac{\left(\begin{array}{c} \text{[Succinyl - Co}{\text{A}}^{4-}{\text{][CO}}_{\text{3}}^{\text{2}-}\text{]}\\ \text{[NAD}{\text{H}}^{\text{2}-}\text{][}{\text{H}}^{\text{ + }}\text{]} \end{array}\right)}{\left(\text{[}\alpha \text{K}{\text{G}}^{\text{2 - }}\text{][CoAS}{\text{H}}^{4-}\text{][NA}{\text{D}}^{-}\text{]}\right)}\right) \end{array}$$

The kinetic expressions for the regulatory effects of various cofactors on OGDHC (which depend on 10 additional kinetic parameters) are parameterized in the Results section, and are estimated separately (see below). Because CO_2, tot _dependent terms are not included in the denominator in Equation (11), CO_2, tot _dependent product inhibition is not accounted for in Equation (12).

### Parameter estimation

The developed kinetic model of OGDHC has 8 adjustable parameters for catalytic mechanism and 10 adjustable parameters for cofactor regulation (Table 1). Parameter values were estimated in a systematic modular manner in multiple steps by least-squares fitting of the model simulated steady-state flux to the available experimental data as detailed in Results section below. The FMINCON algorithm in MATLAB (The MathWorks, Natick, MA) was used to solve this non-linear optimization problem. In addition, sensitivity analysis was performed to estimate the sensitivity of the least square error to small changes in the optimal parameter values. The sensitivity was computed using:

**Table 1:** Kinetic parameter values for 2-oxoglutarate dehydrogenase complex

Basic kinetic parameters			Kinetic parameters for regulatory cofactors		
Parameter:	Value	Sensitivity	Parameter:	Value	Sensitivity
*V*_max _μmol mg^-1 ^min^-1a^	-	-	*K_aCa _*(μM)	0.893	0.293
*K_mA _*(mM)	0.273	3.601	*K_iATP _*(mM)	0.106	0.943
*K_mB _*(μM)	6.96	0.934	*K_iADP _*(mM)	0.305	2.073
*K_mC _*(μM)	98.6	2.839	*K_aMg _*(μM)	19.49	0.228
*K_ia _*(mM)	75.9	1.981	*$\alpha_{Ca}^{0}$*	0.262	9.648
*K_ir _*(mM)	2.4	1.995	*α_ATP_*	6.694	1.628
*K_ic _*(mM)	0.112	3.970	*α_ADP _*	0.173	4.412
*K_iq _*(μM)	0.218	5.224	*α_Mg_*	1.00	6.594
*K_aH _*(pH)	6.11	3.623	*β_Mg_*	4.222	4.603

^a ^We cannot estimate the value of parameter *V_f_*, _max _in the model without knowing the enzyme activity in the experimental setup. The value of *V*_max _reported in the table is equal *X *× *V_f_*, _max_, where *X *is the enzyme activity. The optimization estimates that *V*_max _≈ 2.16 μmol mg^-1 ^min^-1 ^for Figure 2, *V*_max _≈ 11.033 unit/mg for Figure 3 and Figure 4, *V*_max _≈ 167 nmol mg^-1 ^min^-1 ^for Figure 5.

(13) $$S_{i}=\frac{\max\left(\left|E_{i}^{*}(x_{i}\pm 0.1x_{i})-E_{i}^{*}(x_{i})\right|\right)}{0.1E_{i}^{*}(x_{i})},$$

where E* is the least square difference between model simulations and experimental data, and *x_i _*is the optimized value of the *i^th ^*parameter.

Since all kinetic parameters in the model are measured relative to species concentration, we performed a composition analysis to estimate the concentration of all ionic species all experiments analyzed here [36].

## Results

### Parameterization of basic kinetic mechanism of OGDHC

In this section, we present the detailed parameterization and validation of the proposed kinetic model based on the available experimental data on the kinetics of OGDHC, measured in a wide variety of experimental conditions. To study the catalytic mechanism of OGDHC, McMinn and Ottaway [20] investigated the kinetic properties of the OGDHC system, which was prepared from fresh pig heart mitochondria. Following the method of Fromm [21] in which it was concluded from initial velocity studies that the catalytic mechanism of OGDHC is not consistent with the Hexa-Uni-Ping-Pong mechanism. While their observations suggest a random order kinetic mechanism with respect to the binding of NAD^- ^and CoASH^4- ^and release of Succinyl-CoA^4-^, the binding of 2-oxoglutarate and release of CO_2 _is described as a Ping-Pong mechanism. Initial velocity kinetics measured by Smith *et al*. [10] with purified pig heart mitochondria OGDHC showed that Succinyl-CoA^4- ^and NADH^2- ^were inhibitors, but no inhibitory effects were observed with GTP or ATP. Their results also show that Succinyl-CoA^4- ^inhibition was competitive with CoASH^4- ^and independent of the NAD^- ^oxidation-reduction state. These data are used here to identify the kinetic parameters of our OGDHC model.

**Figure 2:**
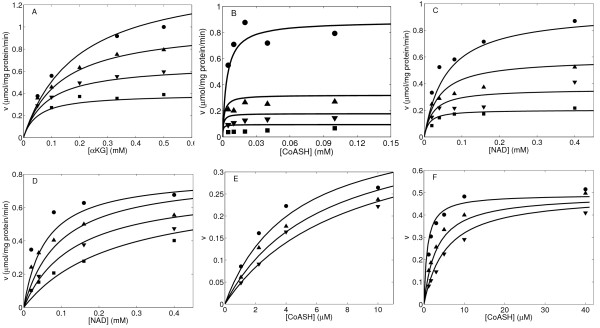
**OGDHC activity as a function of different substrate and product concentrations. ** (A) OGDHC activity as a function of 2-oxoglutarate concentration at different NAD and CoASH concentrations in the absence of products. Kinetic data were obtained from Figure One A of [20]. Concentration of substrates NAD and CoASH in the assay were fixed to: 0.033 mM and 0.005 mM (square); 0.066 mM and 0.01 mM (down-triangle); 0.133 mM and 0.02 mM (up-triangle); 0.333 mM and 0.05 mM (circle). (B) OGDHC activity as a function of CoASH concentration at different 2-oxoglutarate and NAD concentrations in the absence of products. Kinetic data were obtained from Figure One B of [20]. Concentration of the substrates 2-oxoglutarate and NAD in the assay were fixed to: 0.025 mM and 0.02 mM (square); 0.05 mM and 0.04 mM (down-triangle); 0.1 mM and 0.08 mM (up-triangle); and 0.5 mM and 0.4 mM (circle). (C) OGDHC activity as a function of NAD at different 2-oxoglutarate and CoA concentrations in the absence of products. Kinetic data were obtained from Figure One C of [20]. Concentration of the substrates 2-oxoglutarate and CoASH in the assay were fixed to: 0.05 mM and 0.005 mM (square); 0.1 mM and 0.01 mM (down-triangle); 0.2 mM and 0.02 mM (up-triangle); and 0.5 mM and 0.05 mM (circle). (D) OGDHC activity as a function of NAD at different 2-oxoglutarate and CoASH concentrations in the presence of products NAGH. Kinetic data were obtained from Figure Two B of [20] and rescaled for data consistence. Concentration of the substrates 2-oxoglutarate and CoASH in the assay were fixed to: 0.5 mM and 0.05 mM. NADH concentration were 0.0 (circle), 0.01 mM (up-triangle), 0.02 mM (down-triangle) and 0.05 mM (square). (E) OGDHC activity as a function of CoASH concentration in the presence of product Succinyl-CoA. Kinetic data were obtained from Figure Five A of [10]. Concentration of the substrates 2-oxoglutarate and NAD in the assay were fixed to: 1.0 mM and 336 μM. Succinyl-CoA concentration were 0.0 (circle), 6.6 μM (up-triangle), and 13.2 μM (down-triangle). (F) OGDHC activity as a function of CoASH concentration in the presence of products Succinyl-CoA and NADH. Kinetic data were obtained from Figure Five B of [10]. Concentration of the substrates 2-oxoglutarate and NAD in the assay were fixed to: 1.0 mM and 16 μM. NADH concentration was 42 μM. Succinyl-CoA concentration were 0.0 (circle), 6.6 μM (up-triangle), and 13.2 μM (down-triangle). Solid lines are model fitting results from the data points represented by symbols. Experimental data were obtained in 0.08 mM potassium phosphate buffer at pH 7.2 and 30°C in Figures 2A-2D, and in 0.1 mM potassium phosphate at pH 7.2 and 22°C in Figures 2E-2F.

The experimental data in Figure 2 were used to estimate the values of unknown kinetic parameters that govern the basic catalytic mechanism of OGDHC (Equation (12)) based on the best fits of the model to the data (See Figure caption for details). Measured enzyme activity is expressed in μmoles NADH^2- ^formed/mg protein/min. We follow a systematic optimization procedure to estimate each kinetic parameter of the model using appropriate experimental data. In the first step, Figure one of McMinn and Ottaway [20] is used to determine the parameters *K_mA_, K_mB _*and *K_mC_*. Because these data describe the changes in the initial rate of OGDHC activities in response to variations in the concentration of different substrates in the absence of products, they facilitate identifying the kinetic parameter associated with the binding of 2-oxoglutarate, CoASH^4-^, and NAD^- ^. Next, the kinetic data from Figure three of McMinn and Ottaway [20] are used to estimate *K_ia _*and *K_ir_*. Finally, the kinetic results from Figure five of Smith *et al*. [10] are used to determine the last two parameters related to Succinyl-CoA^4- ^inhibition: *K_ic _*and *K*_i *q*_. Henceforth, these kinetic parameters are fixed at their estimated values (Table 1).

### Parameterization of the cofactor-dependent regulatory mechanisms

Denton *et al*. [23] conducted a number of experiments to study the effects of Ca^2+^, pH, and adenine nucleotides on the activity of OGDHC from pig heart mitochondria. Their data are used here to identify the kinetic parameters that characterize the activation/inhibition mechanism of Ca^2+^, pH, and adenine nucleotides (See Figure caption for details). Enzyme activities are expressed as units of enzyme activity per mg of protein. (One unit of activity is the amount of enzyme which transforms 1 μmol of substrate per minute at 30°C).

**Figure 3:**
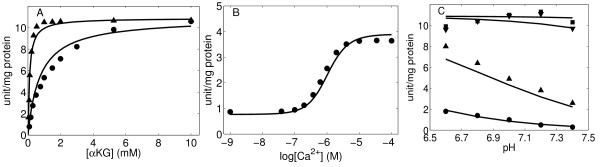
**OGDHC activity regulated by calcium and pH. ** (A) OGDHC activity as a function 2-oxoglutarate concentration with 1.0 mM NAD, 0.25 mM CoASH, and in the presence of 5 mM EGTA (circle) or 5 mM EGTA plus 5 mM CaCl2 (up-triangle)Kinetic data were obtained from Figure Three of [23]. (B) OGDHC activity as a function free calcium concentration in the presence of 1.0 mM NAD, 0.25 mM CoA and 0.1 mM 2-oxoglurate. Kinetic data were obtained from Figure Four of [23]. (C) OGCHC activity as a function of pH in the presence of 1.0 mM NAD, 0.25 mM CoASH with additions of 0.1 mM 2-oxoglurate plus 5 mM EGTA plus 5 mM CaCl_2 _(up-triangle) or plus 5 mM EGTA (circle), or with additions of 25 mM 2-oxoglutarate plus 5 mM EGTA plus 5 mM CaCl_2 _(square) or plus 5 mM EGTA (down-triangle). Kinetic data were from Table 2 in [23]. Solid lines are model fitting results for the data points represented by symbols. Measurements were conducted at pH 7.0 and 30°C for Figure 3A and pH 6.8 and 30°C for Figure 3B and all in 50 mM MOPS buffer plus 1 mM dithiothreitol and with additions of appropriate substrates.

To fit the data that describe the regulatory effects of Ca^2+ ^from Figures 3 and 4 of Denton *et al*. [23], it is necessary to account for mechanisms of allosteric activation/inhibition of Ca^2+ ^in the model. It has been shown that Ca^2+ ^can significantly affect the function of OGDHC by modulating the enzyme affinity for 2-oxoglutarate, showing a sigmoidal kinetics. Here we propose a general scheme of nonessential and mixed-type activation to characterize the effects of Ca^2+^. Based on this scheme, we assume the presence of two binding sites for Ca^2+ ^on OGDHC and modify *V*_max _and *K_m _*of the enzyme complex for 2-oxoglutarate as follows:

**Figure 4:**
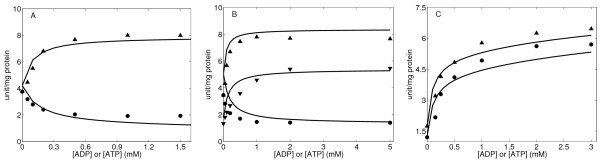
**OGDHC activity regulated by nucleotide with and without calcium** (A, B) OGDHC activity as a function of various concentrations of ATP (circle), ADP (up-triangle) or ADP with 1.5 mM ATP (down-triangle) in the presence of 1.0 mM NAD, 0.25 mM CoASH, with the addition of either 0.1 mM 2-oxoglurate plus 5 mM EGTA plus 5 mM CaCl2 (A) or 2 mM 2-oxoglurate plus 5 mM EGTA (B). Kinetic data were obtained from Figure Five of [23]. (C) OGDHC activity as a function of ADP/ATP ratio in the presence of 1.0 mM NAD, 0.25 mM CoASH and either 0.1 mM 2-oxoglurate plus 5 mM EGTA plus 5 mM CaCl_2 _(circle) or 2 mM 2-oxoglurate plus 5 mM EGTA (up-triangle). Kinetic data were obtained from Figure Six of [23]. Solid lines are model fitting results for the data points represented by symbols.

(14) $$\begin{array}{c} V_{\max,1}=V_{\max,0}\left(\frac{1+\frac{\beta_{Ca}\left[\text{C}{\text{a}}^{\text{2 + }}\right]}{\alpha_{Ca}K_{aCa}}+\frac{\beta_{Ca}{\left[\text{C}{\text{a}}^{\text{2 + }}\right]}^{2}}{{\left(\alpha_{Ca}K_{aCa}\right)}^{2}}}{1+\frac{\left[\text{C}{\text{a}}^{\text{2 + }}\right]}{\alpha_{Ca}K_{aCa}}+\frac{{\left[\text{C}{\text{a}}^{\text{2 + }}\right]}^{2}}{{\left(\alpha_{Ca}K_{aCa}\right)}^{2}}}\right),\;\\ K_{mA,1}=K_{mA,0}\left(\frac{1+\frac{\left[\text{C}{\text{a}}^{\text{2 + }}\right]}{K_{aCa}}+\frac{{\left[\text{C}{\text{a}}^{\text{2 + }}\right]}^{2}}{K_{aCa}^{2}}}{1+\frac{\left[\text{C}{\text{a}}^{\text{2 + }}\right]}{\alpha_{Ca}K_{aCa}}+\frac{{\left[\text{C}{\text{a}}^{\text{2 + }}\right]}^{2}}{{\left(\alpha_{Ca}K_{aCa}\right)}^{2}}}\right). \end{array}$$

The modified flux expression for OGDHC is obtained by substituting Equation (14) into Equation (11). Three adjustable parameters (*α_Ca_, β_Ca_*, and *K_aCa_*) are estimated based on the data from Denton *et al*. [23]; the model fits are shown in Figure 3A-B. For model simulations, the kinetic constants for substrates: 2-oxoglutarate, NAD^-^, and CoASH^4- ^are fixed at their previously estimated values, obtained from data of McMinn and Ottaway [20]. Based on the fits to these data we find that *β_Ca _*is close to one. For simplicity, we fixed *β_Ca _*= 1, meaning that Ca^2+ ^affects only the *K_m _*of 2-oxoglurate, not the *V*_max_. This mechanism is also consistent with the conclusions of Denton *et al*. [23]. (Statistical analysis of different model formulizations supports the validity of the null hypothesis that adding the extra adjustable parameters, *β_Ca_*, does not lead to any significant improvement in fitting results.) The estimated values of the kinetic parameters are summarized in Table 1.

Experimentally it has been shown that the maximal activity of OGDHC is largely unaffected by changes in pH over the range 6.6-7.4, whereas the *K*_m _of the enzyme is markedly altered by pH in this range [23]. In our model, the effect of pH on the OGDHC activity was described based on the observations studies of Denton and colleagues [23]. Here, protons are treated as the essential activators of OGDHC which increase the binding affinity of the enzyme to 2-oxoglutarate. Therefore, the *K_m _*of 2-oxoglurate is modified by multiplying the term *K_aH_/*[H^+^] such that *K*_*mA*, 1 _= (*K*_*mA*, 1_*K*_*aH*_)/[H^+^] (in Equation (15)). Figure 3C illustrates the model fits to the data obtained from Table 2 in McCormack and Denton [23] where the activity of ODGHC was studied under varying pH in both presence and absence of Ca^2+ ^in the buffer.

**Figure 5:**
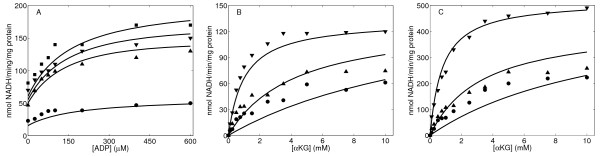
**OGDHC activity regulated by magnesium** (A) OGDHC activity of isolated enzyme as a function of ADP in the presence of 1.0 mM NAD, 0.25 mM CoASH, and 0.5 mM 2-oxoglurate with additions of 0.0 (circle), 25 μM (up-triangle), 50 μM (down-triangle) and 200 μM (square) Mg^2+^. Kinetic data were obtained from Figure Two of [37]. (B-C) OGDHC activity as a function of 2-oxoglutarate in the presence of 1.0 mM NAD, 0.25 mM CoASH with 600 μM (B) or 0 μM (C) Mg^2+ ^addition. (up-triangle) no further additions (control); (circle) +600 μM ATP; (down-triangle) +600 μM ADP. Kinetic data were obtained from Figure Four of [37]. *V*_max _has been changed less than 10% to better fit the symbols. Solid lines are model fitting results for the data points represented by sym-bols. The experimental data were obtained at pH 7.35 and 30°C in 120 mM KCl, 20 mM MOPS-K, 0.5 mM EGTA buffer.

**Figure 6:**
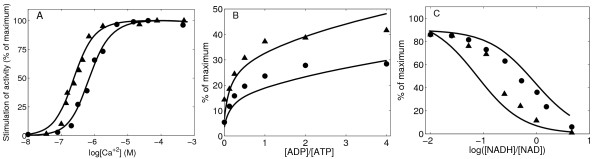
**Predictions of OGDHC activity comparing with experimental results.** (A) OGDHC activity as a function of free calcium concentration in present of 2.0 mM NAD and 0.25 mM CoASH in the presence of either 1.5 mM ATP (circle) or 1.5 mM ADP (up-triangle). Concentrations of 2-oxoglutarate were 0.05 mM (ADP alone) and 0.1 mM (ATP alone). Kinetic data were obtained from Figure Three of [38]. (B) OGDHC activity as a function of ADP/ATP ratio in the presence of 2.0 mM NAD, 0.25 mM CoASH and 0.05 mM 2-oxoglutarate in the presence of either 1.0 nM (circle) or 1.0 μM (up-triangle) free Ca^2+^. Kinetic data were from Figure Four of [38]. (C) OGDHC activity as a function of NADH/NAD ratio 0.35 mM Mg^2+ ^and 0.4 mM total nicotinamide nucleotide in the assay. Ca^2+ ^concentration was either 1 nM (circle) or 0.1 mM (up-triangle) and 2-oxoglutarate concentration was 10 mM (circle) or 0.375 mM (up-triangle). Kinetic data were obtained from Figure Five b of [38]. Solid lines are model predictions for the data points represented by symbols. The experimental data were obtained at pH 7.2 and 30°C in reaction medium of 50 mM MOPS, 75 mM KCl, 60 mM sucrose, 2 mM KH_2_PO_4_, 1 mM EGTA.

Denton and colleagues [23] also studied the effect of adenine nucleotides (ATP and ADP) on the OGDHC activity where it was shown that both ATP and ADP significantly impact the *K*_m _of the en-zyme for 2-oxoglutarate and that the regulations of OGDHC by Ca^2+ ^and adenine nucleotides seem to be independent. Here the regulatory effects of nucleotides on OGDHC activity is modeled as similar to that of Ca^2+^. Specifically, we assume that there are different binding sites on the OGDHC that bind to ATP or ADP. (The available kinetic data cannot exclude the other possibility that ATP and ADP can bind at the same site.) Therefore, the *V*_max _and *K_m _*for 2-oxoglutarate was modified as a function of nucleotide concentrations as follows:

(15) $$\begin{array}{l} V_{\max,2}=V_{\max,1}\frac{\left(1+\frac{\beta_{ATP}{\left[\text{ATP}\right]}_{\text{T}}}{\alpha_{ATP}K_{ATP}}\right)\left(1+\frac{\beta_{ADP}{\left[\text{ADP}\right]}_{\text{T}}}{\alpha_{ADP}K_{aADP}}\right)}{\left(1+\frac{{\left[\text{ATP}\right]}_{\text{T}}}{\alpha_{ATP}K_{ATP}}\right)\left(1+\frac{{\left[\text{ADP}\right]}_{\text{T}}}{\alpha_{ADP}K_{aADP}}\right)},\\ K_{mA,2}=K_{mA,1}\frac{\left(1+\frac{{\text{[ATP}]}_{\text{T}}}{K_{iATP}}\right)\left(1+\frac{{\left[\text{ADP}\right]}_{\text{T}}}{K_{aADP}}\right)}{\left(1+\frac{{\left[\text{ATP}\right]}_{\text{T}}}{\alpha_{ATP}K_{iATP}}\right)\left(1+\frac{{\left[\text{ADP}\right]}_{\text{T}}}{\alpha_{ADP}K_{ADP}}\right)}\frac{K_{aH}}{\left[{\text{H}}^{\text{+}}\right]} \end{array}$$

where [ATP]_T _and [ADP]_T _represent the total concentrations of the nucleotides. Experimental results show that neither free nucleotides nor magnesium binding nucleotides are solely responsible for activation observed. More data are needed to quantitatively and qualitatively specify the activation effects of each nucleotide's ionic forms. Figure 4(A-C) are model fits to the data obtained from Figures 5 and 6 of Denton and colleagues [23]. Optimization results based on these data indicate that both ATP and ADP change the *K_m _*of OGDHC for 2-oxoglutarate without altering the maximum activity, which suggests that both *β_ATP _*and *β_ADP _*are equal to one.

Mg^2+ ^is known to regulate the activity of OGDHC. In a recent study, Rodriguez-Zavala *et al*. [37] examined the effects of ligands, such as ATP, ADP, Ca^2+^, and Mg^2+ ^on the activity of OGDHC in both isolated pig heart enzyme complex and mitochondrial extracts. These data facilitate the characterization of the regulatory effect of Mg^2+ ^on the OGDHC activity and are used here to estimate the Mg^2+ ^associated kinetic parameters. Enzyme activity is measured in nmol NADH^2+ ^formed per minute per mg protein.

Experimental data from purified OGDHC from pig heart mitochondria from Rodriguez-Zavala *et al*. [37] with zero Mg^2+ ^were used to estimate the *V*_max _of the enzyme. Figure 5B shows the model simulations (lines) using the parameter estimates obtained above, with the exception that *V*_max _was adjusted to match these experimental data. Data in Figure 5A with non-zero Mg^2+ ^concentration and the data in Figure 5C are then used to estimate the kinetic parameters associated with the binding of Mg^2+^. Our model fits to these data assume that Mg^2+ ^not only increases the activity by binding to the enzyme complex, but also potentiates the 2-oxoglutarate affinity to the enzyme and decrease the *K_m _*of OGDHC for 2-oxoglutarate. Specifically, we assume two binding sites for Mg^2+ ^and modify the *V*_max _and the *K_m _*as

(16) $$\begin{array}{l} V_{\max,3}=V_{\max,2}\left(\frac{1+\frac{\beta_{Mg}[{\text{Mg}}^{\text{2+}}]}{\alpha_{Mg}K_{aMg}}+\frac{\beta_{Mg}{\left[{\text{Mg}}^{\text{2+}}\right]}^{2}}{{\left(\alpha_{Mg}K_{aMg}\right)}^{2}}}{1+\frac{\left[{\text{Mg}}^{\text{2+}}\right]}{\alpha_{Mg}K_{aMg}}+\frac{{\left[{\text{Mg}}^{\text{2+}}\right]}^{2}}{{\left(\alpha_{Mg}K_{aMg}\right)}^{2}}}\right),\\ K_{mA,3}=K_{mA,2}\left(\frac{1+\frac{{\text{[Mg}}^{\text{2+}}]}{K_{aMg}}+\frac{{{\text{[Mg}}^{\text{2+}}]}^{2}}{K_{aMg}^{2}}}{1+\frac{\left[{\text{Mg}}^{\text{2+}}\right]}{\alpha_{Mg}K_{aMg}}+\frac{{\left[{\text{Mg}}^{\text{2+}}\right]}^{2}}{{\left(\alpha_{Mg}K_{aMg}\right)}^{2}}}\right) \end{array}$$

The data shown in Figure 5 are used to estimate the adjustable kinetic parameters related to Mg^2+ ^ions in our kinetic model for OGDHC. Fits to the data are plotted in Figure 5 and the parameter values summarized in Table 1. The developed model is able to satisfactorily explain the effect of Mg^2+ ^ions on the enzyme activity. These results, combined with those shown in Figures 2 and 3, imply that the matrix free Ca^2+ ^and Mg^2+ ^ions concentrations exert significant and distinct effects on the OGDHC activity.

### Complete flux expression for the 2-oxoglutarate dehydrogenase complex

Based on the proposed mechanisms of allosteric activation and inhibition of various cofactors, the flux expression (Equation (11)) of the OGDHC can be further modified. Applying the catalytic and regulatory mechanisms of Equations 14-16, the final forward flux expression is

(17) $$J_{ogdhc}^{+}=\frac{V_{f}[\text{A][B][C}]\cdot N}{\left(\begin{array}{l} \left[\text{A][B][C}]+\frac{1}{K_{ir}}K_{mC}[\text{A][B][R}\right]\\ +K_{mC}[\text{A][B]+}\frac{1}{K_{iq}}K_{mB}[\text{A][C][Q}]\\ +K_{mB}[\text{A][C}]+\frac{K_{mB}K_{ic}}{K_{iq}K_{ir}}[\text{A][Q][R}]\\ +\frac{K_{ic}}{K_{iq}}K_{mB}[\text{A][Q]}+K_{mA}\text{[B][C}]\alpha_{A}\\ +\frac{K_{ia}}{K_{ir}}K_{mC}\text{[B][R}]+\frac{K_{mB}K_{ic}K_{ia}}{K_{iq}K_{ir}}\text{[Q][R}] \end{array}\right)}$$

where $\alpha_{A}=\frac{\begin{array}{l} \left(1+\frac{\left[{\text{Ca}}^{\text{2+}}\right]}{K_{aCa}}+\frac{{\left[{\text{Ca}}^{\text{2+}}\right]}^{2}}{K_{aCa}^{2}}\right)\times \left(1+\frac{{\text{[ATP}]}_{\text{T}}}{K_{iATP}}\right)\\ \times \left(1+\frac{{\left[\text{ADP}\right]}_{\text{T}}}{K_{aADP}}\right)\times \left(1+\frac{{\text{[Mg}}^{\text{2+}}]}{K_{aMg}}+\frac{{{\text{[Mg}}^{\text{2+}}]}^{2}}{K_{aMg}^{2}}\right) \end{array}}{\begin{array}{l} \left(1+\frac{\left[{\text{Ca}}^{\text{2+}}\right]}{\alpha_{Ca}K_{aCa}}+\frac{{\left[{\text{Ca}}^{\text{2+}}\right]}^{2}}{\alpha_{Ca}^{2}K_{aCa}^{2}}\right)\times \left(1+\frac{{\left[\text{ATP}\right]}_{\text{T}}}{\alpha_{ATP}K_{iATP}}\right)\\ \times \left(1+\frac{{\left[\text{ADP}\right]}_{\text{T}}}{\alpha_{ADP}K_{ADP}}\right)\times \left(1+\frac{\left[{\text{Mg}}^{\text{2+}}\right]}{\alpha_{Mg}K_{aMg}}+\frac{{\left[{\text{Mg}}^{\text{2+}}\right]}^{2}}{\alpha_{Mg}^{2}K_{aMg}^{2}}\right) \end{array}}\times \left(\frac{K_{aH}}{\left[{\text{H}}^{\text{+}}\right]}\right)$

and $N=\frac{\left(1+\frac{\beta_{Mg}\left[\text{M}{\text{g}}^{\text{2 + }}\right]}{\alpha_{Mg}K_{aMg}}+\frac{\beta_{Mg}^{}{\left[\text{M}{\text{g}}^{\text{2 + }}\right]}^{2}}{{\left(\alpha_{Mg}K_{aMg}\right)}^{2}}\right)}{\left(1+\frac{\left[\text{M}{\text{g}}^{\text{2 + }}\right]}{\alpha_{Mg}K_{aMg}}+\frac{{\left[\text{M}{\text{g}}^{\text{2 + }}\right]}^{2}}{{\left(\alpha_{Mg}K_{aMg}\right)}^{2}}\right)}.$

Estimated values of *K_aH_, K_iATP_, K_aADP_, K_aMg_, α_ADP_, α_ATP_, α_Ca_, α_Mg_*, and *β_Mg _*are listed in Table 1.

### Independent validation of the developed kinetic model of OGDHC

Finally, the model is independently validated (corroborated) by comparing the model predictions to the initial rate data of Rutter and Denton [38] on the kinetics of OGDHC obtained from permeabilized mitochondria and mitochondrial extracts (see Figure 6(A-C)). They studied the regulations of NAD-linked isocitrate dehydrogenase and 2-oxoglutarate dehydrogenase by Ca^2+^, nucleotide and nicotinamide nucleotides in permeabilized rat heart mitochondria and in mitochondria extracts. Data from their study were not used for estimation of model parameters and used here to further validate the proposed mechanisms and regulation of OGDHC. Therefore, the flux expression of Equation (17) was used for simulations with the values of the kinetic parameters the same as estimated before (see Table 1). The model accurately describes the kinetics and regulation of OGDHC, observed experimentally, without having to re-estimate the model kinetic parameters, signifying the accuracy of the model and the associated model parameters.

To determine the degree to which the model simulations are sensitive to the estimated parameter values, the relative sensitivities are computed and listed in Table 1. A high sensitivity value indicates that a small change in a given parameter can lead to significant changes in model outputs, used to identify the parameter values. All of our adjustable parameters of the model have sensitivities over 30%. Two parameter estimates (*K_aCa _*and *K_aMg_*) show relatively low sensitivity compared to the others, indicating that predictions of the developed model are less sensitive to these two values. This implies that these two parameters may not be identified accurately by the present analysis, given the sparseness of the data sets analyzed in this work regarding the regulation of OGDHC by Ca^2+ ^and Mg^2+^. Further experiments are required to adequately establish the appropriate regulatory mechanisms and the robustness of each model parameters.

## Discussion

A number of kinetic models have been previously developed to explain the basic catalytic mechanisms and regulations by cofactors of OGDHC. Sanadi *et al*. [12] first proposed a Hexa-Uni-Ping-Pong mechanism for the overall reaction by studying various roles and locations of the cofactors: thiamine pyrophosphate, lipoic acid, and FAD^2- ^within this complex. Hemada *et al*. [19] conducted kinetic studies and proposed a similar mechanism to that of Sandi *et al*. and suggested that NADH^2- ^is a competitive inhibitor of NAD^-^. Whereas Smith *et al*. [10] suggested a noncompetitive inhibition of NADH^2- ^with NAD^-^, the catalytic mechanism was not consistent with Sandi *et al*. Later, McMinn and Ottaway [20] tested a series of possible alternate mechanisms using computer optimization techniques and initial velocity studies and concluded that the binding of NAD^- ^and CoASH^4- ^and the release of Succinyl-CoA^4- ^is a random order, whereas the binding of the substrate 2-oxoglutarate and release of the product CO_2 _still follows a Ping-Pong mechanism. Besides above experimental studies, a number of integrated models of mitochondrial bioenergetics have been developed which used different type of OGDHC models. Cortassa *et al*. [39] describe the activity of OGDHC as a function of Ca^2+^, Mg^2+ ^and substrate concentrations using phenomenological terms. Wu *et al*. [40] used a simple kinetic model of OGDHC from Kohn and Garfinkel [41] in their integrated model of TCA cycle that does not incorporate the regulatory effect of metal ion cofactors. In a recent integrated study of mitochondrial bioenergetics, Bazil *et al*. [42] developed a kinetic model of OGDHC based on a Hexa-Uni-Ping-Pong mechanism with a general description of the cofactor dependency of OGDHC activity. In summary, there have been a wide variety of kinetic models of OGDHC with contrasting kinetic mechanism and cofactor regulations.

In this paper, we developed a unified mechanistic model of OGDHC, in which Ca^2+^, Mg^2+^, ADP^3-^, and pH are treated as activators and ATP^4- ^as inhibitor of the OGDHC activity. The present model offers more realistic and meaningful explanations on the catalytic properties and regulation mechanisms of OGDHC than previous attempts. The analysis also provides a unique set of kinetic parameters that consistently describe a wide variety of experimental data sets on OGDHC function, obtained from diverse sources. Based on the assumed ter-ter mechanism and associated conformational changes, we are able to consistently reproduce the observed kinetics of OGDHC with a minimal number of model parameters. Thus, the proposed mechanism is found to be more appropriate compared to other alternate kinetic models [43].

### Effects of nucleotides on the OGDHC activity

Energy-linked regulators, ADP and ATP, as well as inorganic phosphate, have been investigated for over two decades for their profound effects on kinetic properties of OGDHC. Kinetic studies of mammalian OGDHC, isolated from varied sources, have shown that ADP causes activation of OGDHC [44-47]. This enzyme complex is sensitive to ADP, where ADP significantly decreases the *K_m _*for 2-oxogluterate without affecting the maximum rate (*V*_max_) of the reaction via allosteric interactions. For example, studies on the OGDHC of rat heart mitochondria show a seven fold decrease in the *K_m _*value for 2-oxogluterate by ADP and thereby strongly increases the affinity of OGDHC for the substrate [47]. Other studies of OGDHC from human heart make similar conclusions on the activating effect of ADP [48]. It has also been shown that, at subsaturating concentration of 2-oxoglutarate the relationship between initial reaction rates of OGDHC and concentration of ADP is sigmoidal, suggesting a positive cooperativity in binding of ADP to the enzyme complex [49]. In contrast, at a suboptimal 2-oxoglutarate concentration, ATP was shown to inhibit OGDHC activity in pig heart and bovine kidney mitochondria [23,45]. In addition direct inhibition effect, recent investigations have shown the possible indirect inhibition OGDHC by ATP because of chelation of divalent ions which activate OGDHC, such as Ca^2+^, Mg^2+ ^[37]. The activating action of ADP and inhibiting action of ATP are in competitive opposition (Figure 3E). It is still unclear if these two effectors bind on the same site on the complex or not. Model analysis based on the available kinetic data cannot exclude either possibility. In our mechanistic model, the regulatory effect of ADP and ATP is incorporated by assuming different binding sites for ADP and ATP in the enzyme complex and the model satisfactorily describes the activating effect of ADP and inhibitory effect ATP observed in many experiments [46].

While Zavala *et al*. interpret the data of Figure 5A to indicate that MgADP^- ^is the effective activator of OGDHC activity, our model analysis of the available data sets on Mg^2+^, ADP, and ATP dependent kinetics (Figures 4 and Figure 5B and 5C) reveals that, magnesium and ADP have independent parallel effects on the OGDHC activity, the most parsimonious explanation of the data. However, al-ternative, more complex, models cannot be ruled out.

Like ADP, Pi has also been shown to decrease the *K_m _*value for 2-oxogutarate, without affecting the *V*_max _of OGDHC reaction [44,50]. In a recent report, the Pi activation showed biphasic behavior, with pH dependence [37]. In the physiological concentrations range Pi exerts monophasic activation of OGDHC [37], which can be descried by Equation (15) with three extra parameters. Due to the lack of consistent kinetic data for Pi effects, we do not integrate a Pi dependent regulation mechanism in our current model. However, this energy linked effector may be physiologically important. The overall rate of oxidative phosphorylation is largely determined by phosphorylation potential [51,52]. In cells when ATP utilization increases, the production of ADP and Pi increase. Therefore, activation of OGDHC by ADP and Pi may represent a compensating effect.

### Effect of pH on the OGDHC activity

Mitochondrial matrix proton (H^+ ^ion) concentration is known to affect the OGDHC properties. Specifically, studies on pig heart OGDHC showed that the change in pH in the range 6.6 to 7.4 can significantly alter *K_m _*of the enzyme for 2-oxogluterate, without affecting its maximal activity. McCormack and Denton illustrated the effect of pH on OGDHC activity both in the absence and presence of Ca^2+ ^in their assay mediums [46]. Other experimental observations have shown that hydrogen ions favor the higher affinity of OGDHC for 2-oxoglutarate [45,53]. In our model we hypothesize that hydrogen ions are essential activators of OGDHC activity to describe the observed pH dependency of the OGDHC kinetics.

### Effects of Ca^2+^, Mg^2+^, and EGTA on the OGDHC activity

Studies by McCormack and Denton demonstrate the activating effects of Ca^2+ ^ions on intra-mitochondrial dehydrogenases: pyruvate (PDH), NAD-isocitrate (NAD-ICDH), and 2-oxoglutarate (OGDHC) [24,54,55]. Specifically, the rise in cytosolic Ca^2+ ^concentration in response to extrinsic stimuli, such as hormones can enhance mitochondrial oxidative metabolism via direct activation of these three Ca^2+ ^sensitive dehydrogenases. Such mechanisms may serve as a complementary way to stimulate ATP-synthesis to meet the increased energy demand of the cell [24,54,55].

Mg^2+ ^ion has also been shown to regulate the OGDHC activity either by directly activating the enzyme or by modulating the Ca^2+ ^effects on the enzyme. In some studies, Mg^2+ ^shows no effects on OGDHC activity [23]. However, in other studies, Mg^2+ ^is shown to increase the maximal activity of the enzyme and the affinity of OGDHC for 2-oxogluterate by enhancing the Ca^2+ ^stimulatory effects on the enzyme complex [9,25,37]. These different observations could be accounted for the different levels of endogenous Ca^2+ ^and Mg^2+ ^present in the purified enzyme complex prepared by different methods. Another possible explanation is that the stimulatory effects of Mg^2+ ^is TPP-dependent, which is not explicitly considered in our model. Panov and Scarpa [9] found that Mg^2+ ^only exerts its stimulatory effects in the presence of TPP, though exclusion of TPP from the reaction medium has no effect on the initial enzyme activity in the absence of Mg^2+^. Also, it has been clearly shown that Mg^2+ ^may affect the rate of oxidative phosphorylation in isolated mitochondria primarily via modulating the OGDHC activity [25]. The site of action of Mg^2+ ^ion on OGDHC is unknown. In the present model, we hypothesized a general scheme of nonessential activation of Ca^2+^, by considering two Ca^2+ ^and Mg^2+ ^binding sites on OGDHC. The Mg^2+ ^effect is incorporated in our model by exclusively modifying the enzyme activity and 2-oxoglutarate binding step. So the parameters *V*_max _and *K_mA _*are accordingly expressed as functions of Mg^2+ ^(Equation (16)). Currently, our model assumes that the turn-over rate of E1 is modified to same value for binding either one ion or two ions. And to make it simple, our model does not include possible interaction between Mg^2+ ^and Ca^2+ ^at high concentrations either. The effects are not additive [9] at high concentration, suggesting that Mg^2+ ^and Ca^2+ ^may compete for the binding site. Additional kinetic data set are necessary to test different mechanisms and refine our model to more accurately describe the nature of cation dependent kinetic of OGDHC.

EGTA, which is used in many studies to control Ca^2+ ^ion concentration in reaction media, has been shown in experiments and theoretical analysis to inhibit the NAD-linked isocitrate dehydrogenases (ICDH) through the binding complex, MgEGTA [36]. To date, it is still not clear if there is similar inhibition effect of EGTA or EDTA on the activity of OGDHC. McCormack and Denton [23] concluded that the OGDHC sensitivity toEGTA is very similar to that observed with ICDH [56], because addition of calcium chelators EGTA or EDTA is associated with a marked decrease in the activity of OGDHC at 0.2 mM 2-oxoglutarate. Panov and Scarpa [9], in ascribing the inhibition effect of EGTA to the complex formation between Ca^2+ ^and chelators, concluded that the effected of Ca^2+ ^and chelators is associated with different endogenous cation levels in different preparations. But this explanation cannot account for McCormack and Denton's observation that EGTA or EDTA causes a 40% decrease of activity of OGDHC after using Chelex remove much of the endogenous Ca^2+ ^in the buffer. It is also noted that the *K_M _*for 2-oxoglutarate in the absence of Ca^2+ ^is 4 ± 1.1 mM measured by Panov and Scarpa [9] for commercially available enzyme (Sigma, St. Louis, lot 44H80801), which is almost 15 times the estimated value used in our model (Table 1) and that found by previous workers [19,27]. Only by using the reported *K_M _*of Panov and Scarpa [9], can we reproduce their data using the same mechanism (Equation (17)). In the absence of clear experimental evidence and sufficient data set, our model does not explicitly account for an inhibition effect of EGTA or EDTA.

## Conclusion

Our mechanistic OGDHC model based on a detailed catalytic mechanism successfully provides a single consistent theoretical explanation for many previously unresolved experimental observations on the kinetics and regulations of OGDHC. In particular, it suggests the most plausible physiologically regulations of OGDHC by NAD(H) oxidation-reduction state, the nucleotide phosphorylation potential, pH and various metal ions (Mg^2+ ^and Ca^2+^). As a rise in NADH can reduce the OGDHC flux and thereby provides feedback regulation through the electron transport chain, it is important to ask how NAD oxidation-reduction state and oxidative phosphorylation state exert a coherent regulation of OGDHC in physiological context. Furthermore, how does the OGDHC respond to stimuli via the mitochondrial Ca^2+ ^transport system? Such questions may be addressed by applying the present model in an integrated framework [43] along with other dehydrogenases [36], the oxidative phosphorylation system [57], electron transfer system [58], and cation transport systems [59-61].

## Authors' contributions

FQ conceived the basic idea, collected experimental data set, developed the model, analyzed data and drafted the manuscript. RKP helped to collect and analyze the data and drafted the manuscript. RKD and DAB advised the study and revised the manuscript. All authors read and approved the final manuscript.
